# SOCIAL NETWORK BY ENVIRONMENTAL DESIGN: FACILITATING THERAPEUTIC EFFECT IN LTCF FOR PEOPLE EXPERIENCING DEMENTIA

**DOI:** 10.1093/geroni/igz038.1727

**Published:** 2019-11-08

**Authors:** Farhana Ferdous

**Affiliations:** Assistant Professor, Department of Architecture, College of Engineering and Architecture Howard University, Washington, D.C., United States

## Abstract

The relationship between the physical environment and the prevalence of social interaction have been a core topic of inquiry in environmental gerontology. It has been estimated that around 25 million people worldwide have dementia, and the number will exceed 80 million by 2040. A growing body of literature in the areas of environment-behavior studies shows that the physical environment affects positive behavioral changes, in turn, affecting individual, group and organizational outcomes, but little research has focused on older adults especially those with cognitive impairment by targeting the Dementia Enabling Environment of care facilities. By using non-pharmacological interventions, the purpose of this study is to initiate positive social network among dementia residents and staff by analyzing the spatial configuration of the physical environment and layout in long-term care facilities (LTCF). The findings may give evidence-based design guidelines for future research and design of memory care facilities to promote therapeutic experience for older people experiencing early to moderate stage dementia. Using a 3-stage, multi-method research design such as space syntax, behavior mapping techniques and direct observations, this study objectively measured the spatial configuration of LTCF (physical environment) to evaluate the provision of social interactions (among dementia residents and staff), promote positive health outcomes and healthy living for people experiencing early to moderate stage dementia. This study was able to establish that the architectural layout and environmental design could have a positive and protective effect against dementia in environmental gerontology and geriatrics.

